# Physiological traits of the symbiotic bacterium *Teredinibacter turnerae* isolated from the mangrove shipworm *Neoteredo reynei*

**DOI:** 10.1590/S1415-47572009005000061

**Published:** 2009-09-01

**Authors:** Amaro E. Trindade-Silva, Erik Machado-Ferreira, Marcus V. X. Senra, Vinicius F. Vizzoni, Luciana A. Yparraguirre, Orilio Leoncini, Carlos A. G. Soares

**Affiliations:** Laboratório de Genética Molecular de Eucariontes e Simbiontes, Departamento de Genética, Instituto de Biologia, Universidade Federal do Rio de Janeiro, Rio de Janeiro, RJBrazil

**Keywords:** *Teredinibacter turnerae*, cellulolytic and nitrogen fixing bacteria, antibiotic activity, mangrove shipworm symbiont, *Neoteredo reynei*

## Abstract

Nutrition in the Teredinidae family of wood-boring mollusks is sustained by cellulolytic/nitrogen fixing symbiotic bacteria of the *Teredinibacter* clade. The mangrove Teredinidae *Neoteredo reynei* is popularly used in the treatment of infectious diseases in the north of Brazil. In the present work, the symbionts of *N. reynei*, which are strictly confined to the host's gills, were conclusively identified as *Teredinibacter turnerae*. Symbiont variants obtained *in vitro* were able to grow using casein as the sole carbon/nitrogen source and under reduced concentrations of NaCl. Furthermore, cellulose consumption in *T. turnerae* was clearly reduced under low salt concentrations. As a point of interest, we hereby report first hand that *T. turnerae* in fact exerts antibiotic activity. Furthermore, this activity was also affected by NaCl concentration. Finally, *T. turnerae* was able to inhibit the growth of Gram-negative and Gram-positive bacteria, this including strains of *Sphingomonas* sp., *Stenotrophomonas maltophilia*, *Bacillus cereus* and *Staphylococcus sciuri*. Our findings introduce new points of view on the ecology of *T. turnerae*, and suggest new biotechnological applications for this marine bacterium.

## Introduction

The family Teredinidae is composed of obligate marine woodboring mollusks of wide geographical distribution (Turner, 1966). Nutrition in shipworms is supported by symbiotic association with the cellulolytic/nitrogen fixing bacterium *Teredinibacter turnerae* (Carpenter and Culliney, 1975; Trytek and Allen, 1980; Gallager *et al.*, 1981; Waterbury *et al.*, 1983; Distel *et al.*, 2002b; Lechene *et al.*, 2007). *T. turnerae* is a Gram-negative marine bacterium, requiring 0.3 M NaCl and other salts for optimum growth. Furthermore, this symbiont can use cellulose as the sole carbon source and fix dinitrogen under micro-aerobic conditions. It also requires combined nitrogen in a vigorously aerated culture (Distel *et al.*, 2002b). *T. turnerae* is located *in symbio* in the shipworm's gills, within specialized structures comprised of bacteriocytes, the so called glands of Deshayes (Distel *et al.*, 2002a; Distel, 2003; Lechene *et al.*, 2007). This bacterium is thought to be the sole symbiont in all the Teredinidae family (Waterbury *et al.*, 1983; Distel *et al.*, 1991, 2002b). In addition, closely related symbiont ribotypes within the *Teredinibacter* clade have been described as co-existing inside one sole gill tissue (Distel *et al.*, 2002a; Luyten *et al.*, 2006). *T. turnerae* is the only known bivalve-gill endosymbiont that can be cultured (Sipe *et al.*, 2000; Distel *et al.*, 2002b), and has been shown to present a potential for biotechnological application (Greene and Freer, 1986; Greene *et al.*, 1988, 1989; Griffin *et al.*, 1992; Ahuja *et al.*, 2004; Lim and Haygood, 2004; Xu and Distel, 2004).

A specific *Teredinibacter* symbiont ribotype was characterized as colonizing the gills and gonads of *Bankia setacea*, a large shipworm found in temperate waters (Sipe *et al.*, 2000). In tropical estuaries the Teredinidae *Neoteredo reynei* infests mangrove wood. Besides being the only species of the genus, it is one of the largest members in the family, reaching 1.5 meters in length (Turner, 1966). In the northern coast of Brazil, this shipworm is locally known as “Turu” and it is popularly used for the treatment of certain infectious diseases, as well as for enhancing food supplements (Andrade, 1979). Other marine invertebrates are the source of bioactive compounds, which in many cases were found to be produced by associated bacteria (Piel, 2004). These findings reinforce the need for understanding bacteria/Teredinidae host interactions.

Two pertinent facts are that the gland of Deshayes has been observed in *N. reynei* (DeMoraes and Lopes, 2003) and that cellulolytic/nitrogen fixing bacteria have already been isolated from the gills thereof (Distel *et al.*, 2002b). Nevertheless, the identity of this mangrove shipworm symbiont has not as yet been confirmed by molecular tools. In the present work *N. reynei* symbionts were isolated and characterized, *T. turnerae* antibacterial activity described for the first time, and bacterial variants with new distinctly physiological traits obtained *in vitro*. Aspects of these traits in *T. turnerae* ecology are discussed, our findings suggesting new biotechnological applications for this marine bacterium.

## Materials and Methods

###  Specimens

Adult *N. reynei* were collected at the Coroa Grande mangrove area in Sepetiba Bay (Rio de Janeiro, Brazil). Animals collected from decaying wood were immediately transported to the laboratory in autoclaved vials and aseptically dissected. The gills, gonads, intestines and siphons were individually washed and processed for the isolation of symbiotic bacteria and/or extraction of total DNA and RNA.

###  Symbiont purification and culture

Freshly dissected *N. reynei* gills were individually washed five times in 1 mL of a sterile sea water/distilled water 3:1 solution (SWS) and then homogenized in 500 μL of SWS. Serial dilutions were inoculated in 1 cm diameter tubes containing 2 mL of a semi-solid Shipworm Basal Medium (SBM), supplemented with 0.2% (w/v) agar and 0.5% (w/v) powdered cellulose (Sigmacell 101), but without combined nitrogen, so as to select nitrogen fixing bacteria under proper microaerophilic conditions, as previously described (Waterbury *et al.*, 1983). Tubes were incubated at 30 °C and individual colonies obtained, after streaking the pellicle growth from the highest dilution growth (10^-4^-10^-7^) on 1% (w/v) agar SBM plates supplemented with 0.5% (w/v) Sigmacell 101 and 0.1% (w/v) NH_4_Cl. Purified bacterial cultures were confirmed for cellulose utilization and growth in semi-solid SBM tubes without combined nitrogen. Purified bacteria were plated onto a Basal Medium (BM) (Greene and Freer, 1986) (modified by A.R. Moreira, unpublished data), with the addition of 0.1% (w/v) NH_4_Cl, 0.3 M NaCl and 0.5% (w/v) cellulose (Sigmacell 101) (BMC) or 0.5% (w/v) sucrose (BMS), as specified. Modified BM contained: KCl, 5.36 mM; MgSO_4_.7H_2_O, 7.7 mM; MgCl_2_.6H_2_O, 7.38 mM; CaCl_2_.2H_2_O, 2.72 mM ; HEPES (N-[2-hydroxyethyl]piperazine-N'-[2-ethanesulfonic acid]), 20.5 mM; Solution A, 10 mL; and a trace metal solution, 1 mL; the medium was set to pH 8.0. Solution A consisted of: K_2_HPO_4_.3H_2_O, 0.1 M; Na_2_CO_3_, 0.11 M; and Fe_2_(SO_4_)_3_, 0.75 mM. The trace metal solution contained: H_3_BO_3_, 46.9 mM; MnCl_2_.4H_2_O, 0.11 M; ZnSO_4_.7H_2_O, 0.70 mM; Na_2_MoO_4_.2H_2_O, 0.16 mM; CoSO_4_.7H_2_O, 0.18 mM; and CuSO_4_.5H_2_O, 0.32 mM. All solid media included 1% (w/v) agar. All *T. turnerae* were grown at 30 °C and cultures stored in liquid BMS at -80 °C, after the addition of 25% (v/v) glycerol.

###  Selecting *T. turnerae* variants

A selective growth condition was designed to spontaneously obtain *T. turnerae* variants capable of growing under low-salt concentration conditions, and of using casein as the sole carbon and combined nitrogen source, as previously observed (Ferreira *et al.*, 2001). *T. turnerae* was challenged to grow on a usually non-permissive growth medium with low salt content, by streaking a pure culture of *T. turnerae* CS30 on plates containing a NaCl-free BM sucrose medium (BMS^Ls^, Low salt) overlaid with 5% (w/v) casein in a BM with (BM5Ca) or without (BM5Ca^Ls^) NaCl, following 5-10 days incubation at 30 °C. Each CS30 streak with positive growth was purified on BM media with 0.5% (w/v) casein and 0.3 M NaCl (BMCa). Distinct from original *T. turnerae* isolates, the new bacterial cultures were then able to grow in a low salt medium (NaCl free), by using either casein (BMCa^Ls^, Low salt medium) or sucrose (BMS^Ls^, Low salt medium) as the carbon source. No NH_4_Cl was added to casein-containing media.

###  Nucleic acids extraction

Freshly dissected *N. reynei* tissues were washed five times in 1 mL TE pH 8.0 and then snap-frozen in liquid nitrogen. Up to 500 μL of each *N. reynei* tissue homogenate were mixed with 500 μL of 5% (w/v) sucrose, 100 mM Tris pH 7.5, 600 mM NaCl, 100 mM EDTA and a 1% (w/v) sodium dodecyl sulfate (SDS) solution, and incubated for 30 min at 60 °C. After Proteinase K (0.4 mg mL^-1^) and RNAse A (20 μg mL^-1^) digestion, samples were phenol/chloroform extracted and bulk DNA precipitated, washed with isopropanol/ethanol, and then dried and resuspended in 50 μL of sterile ultra-pure water.

*T. turnerae* total DNA was extracted from cell pellets of 0.5-1 mL of 2-days growth in liquid BMS. Bacterial cells were resuspended in 0.5 mL of 50 mM Tris-HCl pH 8.0 and a 50 mM EDTA solution, and frozen at -20 °C. Frozen cells were incubated at room temperature for lysozyme (1 mg mL^-1^) digestion. After incubation with 100 μL of 1 mg mL^-1^ Proteinase K in 50 mM Tris, 0.4 M EDTA, a 0.5% (w/v) SDS solution and 20 μg of RNAse A for 20 min at 50 °C (Silhavy *et al.*, 1984), samples were phenol/chloroform extracted. The DNA was precipitated, washed, dried and resuspended in 50 μL of sterile ultra-pure water.

Total RNA was extracted from either *N. reynei* gill tissues or bacterial cell pellets from 1 mL of specific *T. turnerae* growth. The RNeasy Protect Mini Kit (Qiagen, Valencia, CA) was used according to manufacturer's recommendations. Snap-frozen gills were homogenized with 100 μL of RNAlater solution (Qiagen, Valencia, CA) and added to 500 μL of a lysis buffer (RNeasy kit, Qiagen). Recovered bulk RNA was stored at -80 °C.

###  16S rRNA gene analysis

*N. reynei* tissues and bacterial total DNA were used as templates for 16S rRNA gene (16S rDNA) PCR amplification. Eubacterial specific primers 27f and 1492r were used (Lane, 1991). The purified 1.4 kbp PCR products were individually digested with *Hae*III, *Alu*I and separated in 8% non-denaturing polyacrylamide gels. These enzymes were verified as discriminative for many Teredinidae symbiont ribotypes available in GenBank (accession numbers AY028398, AF102866, AY150183, AY150184, AY150578 and DQ272300 to DQ272317). In addition, four different PCR reactions with the same DNA template were combined and the purified 16S rDNA PCR products (150-500 ng) sequenced, by using the DYEnamic ET Dye Terminator Cycle Sequencing Kit (Amersham Biosciences, Piscataway, NJ) and the specific eubacterial 16S rRNA primers 27f, 1492r, 338f, 338r, 907f, 907r, 1100f and 1100r (Lane, 1991). Sequences were edited by using the SeqMan program (DNASTARinc package for Windows platform, 1989-1999), and analyzed for identity matching with BlastN.

### *celA* analyses and *T. turnerae* PCR screening

Screening for *T. turnerae* in distinct shipworm tissues was performed by PCR with primers specific for the Teredinidae *Psiloteredo healdi* symbiont *celA* cellulase gene (Freer *et al.*, 2001). The primer set PcelA-f (5'CTGTATCG GCCGAACCACCTG3') and PcelA-r (5'TTGCGTTCC AGTCGTCTTTCA3') was synthesized to amplify the bases 661-1897 of the *celA* locus, this including the putative *celA* promoter region (Freer *et al.*, 2001). The primers celA*-*f (5'CACCCAGGGCAACACTCAACC3') and celA-r (5'GGCGCGGCTTATGGGATTGAC3') amplified the region 1592-4732 including the entire *celA* ORF. PCR controls were performed with the primer set EukA/EukB for the termini of the eukaryotic 18S rRNA (Medlin *et al.*, 1988; Sipe *et al.*, 2000).

*N. reynei* tissues and *T. turnerae* total RNAs were used as templates for *celA* RT-PCR reactions by means of the SuperScript III One Step RT-PCR System with Platinum^®^*Taq* DNA Polymerase (Invitrogen, Carlsbad, CA) and the primer set celA-f/PcelA-r.

###  Carboxymethylcellulose (CMC) digestion test

*T. turnerae* grown on BMS plates (two days at 30 °C) were streaked onto fresh BMS plates overlaid with 1% (w/v) agar plus 0.1% (w/v) CMC, and incubated for two days at 30 °C. Plates were stained with 1 mg mL^-1^ Congo-red at room temperature for 15 min and then washed with 1 M NaCl for visualization of CMC digestion (Teather and Wood, 1982).

### *T. turnerae* growth kinetics

*T. turnerae* clumps when cultured in liquid medium, thus impairing proper colony counts by regular plating methodology. Therefore, bacterial growth in liquid media (BMC, BMC^Ls^, BMCa or BMCa^Ls^) was quantified by determining the total DNA concentration in the cultures. All pre-inocula were obtained by thoroughly resuspending a loopful of a two days BMS plate growth of the bacterial variant in 28 mL of fresh BMS. Initially, this mix was split into 2 mL aliquots. After two days of growth at 30 °C under 115 rpm, two tubes were used to determine DNA concentration, thereby yielding the “time zero” point determination for the kinetics curves. The entire cell content of the remaining tubes was individually pelleted (9,200 g for 2 min) and used as a pre-inoculum for a fresh 25 mL of the specific tested medium (BMC, BMC^Ls^, BMCa or BMCa^Ls^) in 125 mL erlenmeyers. These cultures were incubated at 30 °C, under 115 rpm, in order to generate duplicated three time-point growths for both NaCl-free and NaCl added conditions. Individual growth flasks were used for each time-point at two, four and eight days of incubation. All assays were performed twice to six times for each time-point and the DNA concentration determined in triplicate for each sample.

Total DNA concentration in each culture was quantified by pelleting the cell content, centrifuging the total volume of the culture at 9,200 g for 2 min, and then washing any adhered cell biofilm with SWS. The cell pellet was resuspended in 5 mL of 50 mM Tris-HCl pH 8.0, 50 mM EDTA solution, and then frozen at -20 °C. Frozen cells were kept at room temperature with 100 μL of 10 mg mL^-1^ lysozyme in 0.25 mM Tris pH 8.0 until melted, and were then kept on ice for 45 min. Proteinase K was added [500 μL of a 1 mg mL^-1^ solution in 50 mM Tris, 0.4 M EDTA, 0.5% (w/v) SDS] and incubated for 20 min at 50 °C. For each culture cell lysate, three 500 μL samples were collected for total DNA purification. RNAse A was added (20 μg mL^-1^) and the extraction processed as described above. Purified DNA was resuspended in 500 μL of ultra pure-water and quantified by A_260nm_ readings. DNA concentrations are shown as μg DNA mL^-1^ of the culture. Differences in the mean number of the log of bacterial DNA concentration in the media were determined by the Student *t-*test, with a *p* value < 0.05 being considered statistically significant.

###  Assessment of *T. turnerae* antimicrobial activity

Plating tests to detect *T. turnerae* antibacterial activity were undertaken by streaking the original symbiont strain CS30 (see Results for details) on plates of Luria-Bertani (LB) media overlaid with BMS top-agar inoculated with bacterial suspensions. A similar top-agar on Sabouraud plates was used for tests of yeast growth inhibition (Kreger-van Rij, 1984). Distinct Gram-negative bacteria families were tested, including *E. coli* DH5α, *Vibrio harveyi* BB120 (Surette and Bassler, 1998), *Pseudomonas putida* (ATCC 15175), *P. fluorescens* (ATCC 13525), *Chromobacterium violaceum* CV026 (Throup *et al.*, 1995), *Sphingomonas* sp. CS81, environmental strains of *Serratia marcescens* CS265, *Stenotrophomonas maltophilia* CS266 and the Gram-positive environmental strains of *Bacillus cereus* CS262 and *Staphylococcus sciuri* BB20-06. *Sphingomonas* sp. CS81 is closely related to *Sphin. panni* (Busse *et al.*, 2005), and was obtained as a pinkish laboratory contaminant in BMS media, its identity being confirmed by 16S rRNA gene sequencing (GenBank EU684539). All the environmental strains were previously isolated in our lab from tick samples and had their identity confirmed by 16S rRNA gene analyses as indicated (GenBank EU693533, EU693532, EU693531 and EU693530, respectively). The yeasts *Saccharomyces cerevisiae* NRRL Y-12632 (ARS Culture Collection NRRL, USDA) and *Candida albicans* NRRL Y-12983 (ARS Culture Collection NRRL, USDA) were also tested. Tested bacteria were initially grown overnight on LB agar. Only *Sphingomonas* sp. CS81 was grown on BMS and tested on both BMS and LB BMS top-agar. Yeasts were grown on Sabouraud agar. All inocula testing was done by individually resuspending a loop-full of each microorganism growth in 4 mL of BMS top-agar.

Crude methanolic extracts of 3-day-old liquid cultures of CS30 in BMS were also tested for antibiotic activity. *T. turnerae* was grown in 100 mL of a liquid medium, the whole culture content then being lyophilized and resuspended in 10 mL of methanol. After filtration of insoluble debris, the extracts were dried by vacuum centrifugation and finally resuspended in 1 mL of methanol. Sterile filter-paper discs soaked with 5 μL of these crude methanolic extracts were air-dried and layed onto plates inoculated with the target bacteria on top-agar. Test plates were incubated for 1-7 days until inhibition-halo detection. All growths were performed at 30 °C, so that all microorganisms were able to grow under the tested conditions.

## Results

###  Isolation and molecular characterization of *N. reynei* symbionts

**Figure 1:**
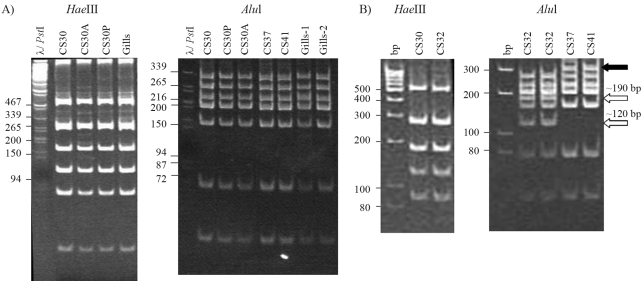
*Hae*III and *Alu*I digestion patterns of 16S rDNA PCR products amplified from cellulolytic/nitrogen fixing bacteria isolates and *N. reynei* gills. (A) Digestion profiles for the *T. turnerae* CS30, CS37, CS41 isolates, gill-bulk DNA amplicons and CS30A and CS30P variants. (B) Distinction of the *T. turnerae* CS32 isolate16S rDNA digestion pattern from CS30, CS37 and CS41 isolates. *DNA markers are shown in base pairs. **Absent and emerging bands in the CS32 profile are indicated by black and white arrows, respectively. ***See Materials and Methods for details.

Bacteria were isolated from the gills of *N. reynei* after selection in cellulose/combined nitrogen-free semi solid SBM medium. Eighteen pure morphologically identical cultures were obtained. The isolates denominated CS30, CS32, CS37 and CS41 were purified from distinct *N. reynei* individuals and randomly selected for further analysis. These bacteria were able to grow in regular BMC or BMS media. 16S rDNA analysis was performed by using each bacterial isolate and *N. reynei* gills bulk DNA. 16S rDNA 1.4 kbp PCR amplicons were individually digested with *Hae*III and *Alu*I (Figure 1). All isolates and the 16S rDNA directly amplified from *N. reynei* gills showed an identical *Hae*III digestion pattern. Only 16S rDNA of CS32 presented a distinct *Alu*I digestion profile. The *Hae*III and *Alu*I patterns observed for CS30, CS37 and CS41 16S rDNAs were identical to those expected for the *T. turnerae* T7902 type strain (GenBank AY028398), thereby indicating that in *N. reynei* the bacterium *T. turnerae* is found as a symbiont, as it is in many other Teredinidae. In fact, the 16S rDNA 1.4 kbp PCR product of both CS30 (chosen as a representative of the *N. reynei* symbiotic consortia) and CS32 was sequenced (GenBank AY949835 and AY949836, respectively). These sequences shared 99% identity with the *T. turnerae* type strain 16S rRNA sequence, thus confirming their being *T. turnerae*. The CS32 16S rDNA sequence presented one additional T-G transversion at position 456 bp, thereby creating an extra *Alu*I restriction site and generating the unique *Alu*I pattern (Figure 1B).

The 16S rDNA PCR products from CS37, CS41 and from the bulk DNA of *N. reynei* gills were also partially sequenced by using the primers 27f, 338r and 907r. These primers flank the variable portions V1, V2 and V3 of the 16S rRNA (Neefs *et al.*, 1993; Van de Peer *et al.*, 1996a, 1996b), and together can discriminate 16S rRNA sequences from shipworm symbiont ribotypes deposited in GenBank (Sipe *et al.*, 2000; Distel *et al.*, 2002a, 2002b; Luyten *et al.*, 2006) (data not shown). Partial sequences from all the samples attributed the highest identity to the *T. turnerae* type strain16S rRNA.

###  Molecular screening for *T. turnerae* in distinct host tissues

**Figure 2:**
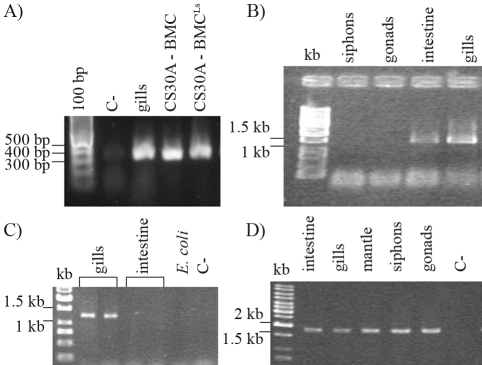
*celA* gene and eubacterial 16S rDNA amplifications from *T. turnerae* and *N. reynei* tissue samples. (A) RT-PCR assessment of *celA* expression in the gills and by *T. turnerae* CS30A growing in cellulose media with 0.3 M NaCl (BMC) or under low salt concentration (BMC^Ls^). Reactions using the primer set celA-f and PcelA-r. (B) PCR for eubacterial 16S rDNA in *N. reynei* tissues. (C) PCR for the *T. turneraecelA* promoter region in *N. reynei* tissues containing eubacteria. (D) PCR amplification of eukaryotic 18S rDNA in *N. reynei* tissue samples.

PCR strategy was used to determine *T. turnerae* distribution in *N. reynei* tissues. Primers to the multidomain cellulase *celA* gene were designed and the presence of *celA* in CS30 was confirmed by both PCR and sequence analysis. A ~4 kbp amplicon, including the entire *celA* gene and its promoter region, was amplified with the PcelA-f/celA-r primer set. NESTED-PCRs, together with the internal primer sets PcelA-f/PcelA-r for the putative *celA* promoter, celA-f/celA-r for the whole *celA* coding sequence and celA-f/PcelA-r, amplified the 1236 bp, 3140 bp and 305 bp products, respectively. These represented the expected amplicons for the described *celA* locus (Freer *et al.*, 2001). Partial sequencing of the 3140 bp fragment confirmed it as a *N. reynei* symbiont *celA* gene version (data not shown). Positive RT-PCR amplification showed that *celA* was being expressed in CS30, and reactions using *N. reynei* gill-bulk RNA indicated this was also so *in symbio* (Figure 2A).

DNA extracts from *N. reynei* gills, gonads, mantle (not shown), siphons and intestine were initially screened for the presence of eubacterial 16S rDNA (Figure 2B). PCR reactions with samples of the gills and intestines generated the expected amplicons, and so were subjected to a specific PCR screening for *T. turnerae* by using putative *celA* promoter primers (Figure 2C). Only the gills produced the expected *celA* band, thereby indicating that the *T. turnerae* symbiont is restricted to this tissue, whereas other eubacteria are present in shipworm intestines. Reactions with gonad, mantle and siphon samples were negative for *celA* (data not shown) and positive for the 18S rDNA controls (Figure 2D).

### *T. turnerae* spontaneous variants

*T. turnerae* is described as a restricted marine bacterium (Distel *et al.*, 2002b), and despite secreting protease(s), it cannot grow by using casein as the sole carbon and nitrogen source (Greene *et al.*, 1989; Griffin *et al.*, 1992). It was also reported to be highly polymorphic (Waterbury *et al.*, 1983; Ferreira *et al.*, 2001; Distel *et al.*, 2002b), and could potentially lead to new emerging physiological traits in this biotechnologically relevant bacterium. In fact, Ferreira *et al.* (2001) reported new physiological traits in a *T. turnerae* variant spontaneously obtained *in vitro*, which appeared as an “aggregate form” with a distinct exopolysaccharide content, and as also having acquired the ability to use casein without the addition of NaCl. However, this unidentified variant was incapable of consuming cellulose. Based on this knowledge, a selective condition was designed to test the ability to select variants from the *N. reynei* symbiont CS30. Two spontaneous *T. turnerae* variants were successfully obtained after selection in a low salt-content medium with casein (*Materials and Methods*). Purified cultures were obtained from those few CS30 streaks with positive growth. One variant presented a yellow color on BM5Ca top agar and was denoted CS30A. The other variant, manifesting intense protease activity and with an evident casein degradation halo on the BM5Ca^Ls^ top agar, was named CS30P. The identity of CS30A and CS30P as *T. turnerae*, besides the absence of any other bacterial type, were confirmed by *Hae*III and *Alu*I 16S rDNA PCR-RFLP (Figure 1A) and partial sequencing of a ~ 800 bp PCR amplicon including the V1 and V2 16S rDNA regions (data not shown).

CS30A and CS30P were still capable of using cellulose and manifest pellicle growth in the combined nitrogen-free/microaerobic conditions of a semi-solid SBM medium. These cultures preserved the same growth ability of the original CS30 isolate, this including cellulase secretion on BMS, as defined by CMC degradation tests (data not shown). However, they had acquired novel physiological traits (Table 1). Plate tests on BMC and BMCa agar, with and without the addition of 0.3 M NaCl, demonstrated that CS30A and CS30P presented vigorous growth when using casein as the sole carbon and nitrogen source, independent of the addition of NaCl. These bacteria were also capable of using cellulose in the absence of NaCl, although *T. turnerae* growth was notably reduced on cellulose agar under these conditions. This specific capacity for growth in these variants was clearly stable, even after having remained for months in SBM. Notably, a direct inoculum of CS30 was incapable of growing either in casein or low-salt medium.

**Figure 3:**
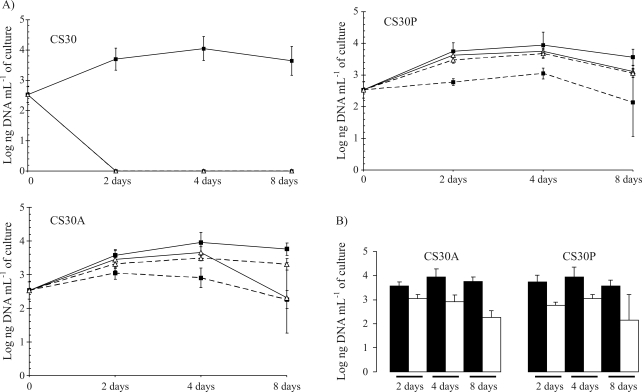
Growth of *T. turnerae* in distinct media. (A) Growth kinetics of the *T. turnerae* original isolate CS30 and the variants CS30A and CS30P, when using cellulose (BMC medium; black squares) or casein (BMCa medium; white triangules) with 0.3 M NaCl (full lines) and in low salt media (dashed lines). *Growth is presented as the Log of the total DNA concentration (ng ml^-1^ of culture) after 2, 4 and 8 days growth at 30 °C and 115 rev min^-1^. **See Materials and Methods for details; (B) Detail showing CS30A and CS30P growth profile, when using cellulose as the sole carbon source with 0.3 M NaCl (black) and in low salt media (white).

Growth kinetics in CS30, CS30A and CS30P is very similar when using cellulose with 0.3 M NaCl, as determined by total DNA concentration in the cultures (Figure 3A). On the other hand, growth kinetics in CS30A and CS30P was similar when using casein or cellulose in a low-salt medium. A more intense growth was observed during the first two days, reaching maximum counts after four days. Although still able to grow under low [Na^+^Cl^-^] conditions, *T. turnerae* variants were clearly constrained thereat. This effect was more pronounced when cellulose was used as the sole carbon source, whereby the low-salt concentration caused a notable drop in *T. turnerae* growth (p < 0.05) (Figures 3A, -3B). Interestingly, *T. turnerae* still maintained *celA* gene transcription, even when grown under these conditions (Figure 2A).

### *T. turnerae* displays antibacterial activity

In order to verify those antimicrobial activities of *N. reynei* symbionts that could potentially be related to its use as a therapeutic, natural-product, direct plating tests were undertaken by growing CS30 on BMS top-agar containing a variety of Gram-negative and Gram-positive bacteria, as well as yeasts. Inhibitory activity could be detected against the Gram-negative strains of *Sphingomonas* sp., *Sten. maltophilia* and the Gram-positive *B. cereus* and *Staph. sciuri* (Table 1, Figure 4). The same result was observed when CS30 crude methanolic extracts were used instead of live bacteria. Direct plating of the *T. turnerae* variants CS30A and CS30P also caused intense inhibition of *Sphingomonas* sp. but had no evident effect on *Sten. maltophilia*, *B. cereus* or *Staph. sciuri* cultures (Table 1). All this indicates that *T. turnerae* potentially secretes various compounds, thereby distinctly inhibiting the growth of *Sphingomonas* and other bacteria. Interestingly, when CS30A or CS30P were tested on the low-salt medium BMS^Ls^, the inhibition of *Sphingomonas* was no longer observed, this indicating that NaCl is required for the production, activity and/or sensitivity of *Sphingomonas* to *T. turnerae* bioactive compounds. A distinct and opposite activity of *T. turnerae* growths and extracts was also detected. This activity was characterized by growth enhancement of tested bacteria observed just beyond the inhibition zone (Figure 4). This growth enhancing activity was investigated no further. In conjunction these data present a new potential application of *T. turnerae* as a bioactive compound producer.

## Discussion

The Teredinidae and their bacterial symbiont *T. turnerae* have attracted interest due to their economic relevance and by offering a unique system for nitrogen fixing bacteria/animal host interaction studies. *N. reynei* is particularly conspicuous through being the biggest member of the Teredinidae family, its role in mangrove ecology and its therapeutic use on the northern coast of Brazil. In the present work, we isolated, characterized and identified *T. turnerae* as its symbiotic bacteria. Two distinct *T. turnerae* strains, represented by the CS30 and CS32 isolates, were found by 16S rDNA analysis of cellulolytic/nitrogen fixing isolates from *N. reynei* gills (Figure 1). 16S rDNA sequencing showed that these two strains differ by a single base pair and are closely related to the *T. turnerae* type strain (Distel *et al.*, 2002a, 2002b). CS30 and CS32 were isolated from distinct gills samples, although at present we do not know whether distinct symbiont ribotypes co-exist in the same individual *N. reynei* host, as was described for *L. pedicellatus* (Distel *et al.*, 2002a; Luyten *et al.*, 2006). However, the CS32 16S rDNA profile was not observed when using gill bulk DNA (data not shown), thereby indicating that this might be a less commonly represented symbiont in *N. reynei*.

Symbiont PCR screening of *N. reynei* gills, gonads, mantle, siphons and intestines strongly suggested that *T. turnerae* is strictly confined to the host's gills (Figure 2). Other eubacteria are present in *N. reynei* intestines, these possibly being associated with filtered particles, since there is no evidence indicating their role in host biology. No eubacteria were detected in mantle, siphon or gonad, thereby giving rise to pertinent questions regarding vertical transmition of symbionts in *N. reynei*.

In the present work, novel *T. turnerae* variants were spontaneously obtained *in vitro,* after challenging the CS30 strain to grow on a usually non-permissive medium with low-salt concentration and casein. It is known that some bacteria have developed clonal expansion strategies to face changing environments (Moxon *et al.*, 1994), and enhancing the probability of survival (Oliver *et al.*, 2000; Bayliss *et al.*, 2001; Giraud *et al.*, 2001). Clonal variants were also observed in other symbionts, such as the nematode γ-proteobacterium *Photorhabdus luminescens*, and were shown to be associated with major genomic variation, including re-arrangements in antibiotic biosynthetic genes (Gaudriault *et al.*, 2008). Interestingly, *T. turnerae* variants presented distinct antibiotic activity (Table 1), although the molecular basis and stimulus to produce these variants are still unknown.

The identity of the purified variants CS30A and CS30P was confirmed as *T. turnerae* by 16S rDNA analysis. They differ from the original isolate by the ability to grow on casein or to use cellulose when subjected to the reduced Na^+^ and Cl^-^ ion concentrations of the basal medium (~ 0.01 to 0.04 M NaCl). This allowed us to show that NaCl is important for optimal *T. turnerae* growth, when using cellulose as substrate (Figure 3, Table 1). This is in agreement with the maximum activity of *T. turnerae* cellulases under 0.2 to 0.4 M NaCl (Greene *et al.*, 1988), and their increased binding activity to cellulose substrate when 0.5 M NaCl was added (Imam *et al.*, 1993). It was shown that the CS30A *celA* gene is expressed even without the addition of NaCl. Nevertheless, further experiments should be carried out to determine the regulation of cellulase gene expression on various salt concentrations. Interestingly, certain Teredinidae, such as *Psiloteredo healdi*, can be found in freshwater (Distel, 2003; Santos *et al.*, 2005). In this case, the intracellular environment in the host's gills may not be affected. Furthermore, there are no reports on the effects of symbiotic cellulase activity on host growth in fresh water.

The isolation of variants with low NaCl requirements contrasts with the original description of *T. turnerae* as being an obligate marine bacterium, with a higher need for NaCl (0.1 to 0.6 M) (Greene and Freer, 1986; Distel *et al.*, 2002b). It is intriguing that, even though *T. turnerae* is able to grow *in vitro* in the absence of a host-cell partnership, a free-living form of this bacterium has never been observed in nature. We have been unsuccessful in attempts to isolate *T. turnerae*, and by means of PCR, detect its *celA* gene in mangrove substrates, this including water, mud, decaying wood, submerged mangrove leaves and tree pneumatophores (data not shown). Previous attempts to isolate free-living forms of *T. turnerae* have also failed (Waterbury *et al.*, 1983). There is the possibility of spontaneous variants of *T. turnerae* arising in nature, which, through novel physiological skills might possibly be capable of colonizing a broad spectrum of aquatic habitats. It is possible that like traits in *T. turnerae* variants could support free-living bacterial populations subjected to the spatial/temporal physicochemical changes that occur in estuarine waters, where salinity varies from zero to over 35 (~ 0.6 M NaCl).

The observed bactericidal activity could be advantageous for *T. turnerae* both in competiting with other bacteria if present as a free-living form in marine environments, or in playing a role *in symbio*. This seems to be a common feature of *T. turnerae*, since another strain isolated in our lab from the shipworm *Lyrodus massa* presented the same antibacterial activity (data not shown). In fact, *Sphingomonas* spp., *Sten. maltophilia*, the Gram-positive bacteria *Bacillus* spp. and *Staphylococcus* spp. are all present in marine environments and associated with invertebrates (Faghri *et al.*, 1984; Cavicchioli *et al.*, 1999; Furushita *et al.*, 2005; Miao and Qian, 2005; Li *et al.*, 2007; Romanenko *et al.*, 2007, 2008; Muscholl-Silberhorn *et al.*, 2008; Zhu *et al.*, 2008). *Sphingomonas* sp. was the only tested bacterium inhibited in the same manner by both the original *T. turnerae* isolate as well as its variants, in an activity which is potentially unrelated to the inhibition of the other tested Gram-negative and Gram-positive bacteria. It is noteworthy that bacteria of the genus *Sphingomonas* present glycosphingolipids in the outer membrane which act as regulatory molecules (Olsen and Jantzen, 2001; Yabuuchi *et al.*, 2002; Heung *et al.*, 2006). Hence it is possible that the bioactive compounds produced by *T. turnerae* might, in turn, affect these molecules. This is being investigated. Moreover, it is conceivable that the systemic spread of bioactive compounds secreted by *T. turneraein symbio* might be related to the popular therapeutic applications of *N. reynei* in the north of Brazil. Further attempts should focus on the characterization of these symbiotic bioactive compounds and the identification of their biosynthetic gene(s).

## Figures and Tables

**Table 1:** *T. turnerae* variants growth and antibiotic activity profile.

*T. turnerae* variant	Growth profile*						Antibiotic activity^†^			
	BM (0.3 M NaCl)			BM^Ls^ (low salt)						
	Cellulose	Casein		Cellulose	Casein		*Sphingomonas* sp.	*Sten. maltophilia*	*B. cereus*	*Staph. sciuri*
CS30	+	-		-	-		+	+	+	+
CS30A	+	+		±	+		+	-	-	-
CS30P	+	+		±	+		+	-	-	-

*Four days growth on BM or BM^Ls^ NaCl-free solid media at 30 °C. “+” = positive growth; “±” = weak growth; “-” = negative growth. Cellulose as the sole carbon source + 0.1% NH_4_Cl or casein as the sole carbon and nitrogen source. ^†^Antibiotic activity of *T. turnerae* variants streaked on BMS or LB plates overlaid with BMS top-agar containing the tested Gram-negative (*Sphingomonas* sp. CS81 or *Stenotrophomonas maltophilia*) or Gram-positive (*Bacillus cereus* or *Staphylococcus sciuri*) bacteria. “+” = *T. turnerae* inhibits the tested bacterium; “-” = *T. turnerae* does not inhibit the tested bacterium. See Materials and Methods for details.
